# The impact of sarcopenia on the incidence of postoperative outcomes following spine surgery: Systematic review and meta-analysis

**DOI:** 10.1371/journal.pone.0302291

**Published:** 2024-08-26

**Authors:** Mingjiang Luo, Zubing Mei, Siliang Tang, Jinshan Huang, Kun Yuan, Lingling Jiang, Zhifeng Tang, Keni Li, Mingxuan Su, Can Su, Yuxin Shi, Zihan Zhang, Jiang Chen, Yuan Zheng, Peng Bin, Zhengbing Yuan, Guosong Xu, Zhihong Xiao

**Affiliations:** 1 The Second Affiliated Hospital, Hengyang Medical School, University of South China, Hengyang City, Hunan Province, China; 2 Department of Anorectal Surgery, Shuguang Hospital Affiliated to Shanghai University of Traditional Chinese Medicine, Shanghai, China; 3 Anorectal Disease Institute of Shuguang Hospital, Shanghai, China; 4 Hengyang Medical School, University of South China, Hengyang City, Hunan Province, China; 5 Department of Pediatric Dentistry, First Affiliated Hospital (Affiliated Stomatological Hospital) of Xinjiang Medical University, Urumqi, China; 6 Department of Orthopaedics, Dongguan Qiaotou Hospital, Dongguan, Guangdong, China; 7 Department of Orthopaedics, The First Hospital of Putian City, The School of Clinical Medicine, Fujian Medical University, Putian, Fujian, China; University of Bologna - Rimini Campus: Universita degli Studi di Bologna - Campus di Rimini, ITALY

## Abstract

**Purpose:**

Sarcopenia is considered to be an important predictor of adverse outcomes following spinal surgery, but the specific relationship between the two is not clear. The purpose of this meta-analysis is to systematically review all relevant studies to evaluate the impact of sarcopenia on spinal surgery outcomes.

**Methods:**

We systematically searched PubMed, Embase and the Cochrane Library for relevant articles published on or before January 9, 2023. The pooled odds ratio (OR) with 95% confidence intervals (CIs) was calculated in a random effects meta-analysis. The main outcome was the risk of adverse outcomes after spinal surgery, including adverse events and mortality. This systematic review and meta-analysis was conducted following the PRISMA guidelines to evaluate the impact of sarcopenia on spinal surgery outcomes. In addition, we also conducted a subgroup analysis and leave-one-out sensitivity analyses to explore the main sources of heterogeneity and the stability of the results.

**Results:**

Twenty-four cohort studies, with a total of 243,453 participants, met the inclusion criteria. The meta-analysis showed that sarcopenia was significantly associated with adverse events (OR 1.63, 95% CI 1.17–2.27, P < 0.001) but was no significantly associated with mortality (OR 1.17, 95% CI 0.93–1.46, P = 0.180), infection (OR 2.24, 95% CI 0.95–5.26, P < 0.001), 30-day reoperation (OR 1.47, 95% CI 0.92–2.36, P = 0.413), deep vein thrombosis (OR 1.78, 95% CI 0.69–4.61, P = 0.234), postoperative home discharge (OR 0.60, 95% CI 0.26–1.37, P = 0.002) and blood transfusion (OR 3.28, 95% CI 0.74–14.64, P = 0.015).

**Conclusion:**

The current meta-analysis showed that patients with sarcopenia have an increased risk of adverse events and mortality after spinal surgery. However, these results must be carefully interpreted because the number of studies included is small and the studies are significantly different. These findings may help to increase the clinicians’ awareness of the risks concerning patients with sarcopenia to improve their prognosis.

## Introduction

Sarcopenia is a skeletal muscle disease that progresses with age and involves accelerated loss of muscle mass and function, including osteoporosis, fall proneness, weakness, dysfunction and death [1]. The occurrence of sarcopenia is related not only to age but also to a series of long-term diseases. Its causes mainly include muscle anabolism disorder, inflammation, malnutrition, insulin resistance, mitochondrial dysfunction, oxidative stress and so on [2]. Clinical data show that sarcopenia is associated with a higher incidence of postoperative complications, reoperation, longer hospital stay, and perioperative morbidity and mortality [3–6]. It usually occurs during the life course of elderly individuals.

In recent years, due to COVID-19 and the increase in the proportion of aging adults worldwide, the prevalence of sarcopenia has increased [7]. Concurrently, there has been an increased frequency of combined cases of spinal disease and sarcopenia. Spinal diseases primarily affect the elderly population and can result in trunk and/or limb pain, paralysis, and/or deformities, ultimately disrupting their motor function. Spinal surgery is a common treatment for most spinal diseases [8], and the factors affecting its prognosis have always been one of the main research directions of spinal surgery. Studies have shown that sarcopenia has been increasingly recognized as an important predictive factor for adverse outcomes following complex spinal surgery [9]. Therefore, there is a need for further analysis and evaluation of the predictive value of sarcopenia for adverse postoperative outcomes of spinal surgery to enable clinical practitioners to identify patients at risk of experiencing adverse outcomes following spinal surgery and implement early interventions to reduce the incidence of postoperative adverse events.

An increasing number of studies have begun to focus on the effects of sarcopenia on spinal surgery patients. Zakaria, H.M. et al. found that sarcopenia can predict postoperative mortality, adverse events and infection in patients with spinal metastases who undergo thoracolumbar revision surgery [4, 5]. In addition, Hirase, T. et al. reported that sarcopenia can also predict the incidence of deep venous thrombosis in thoracolumbar revision surgery patients [5]. However, some studies have found different conclusions. Barile, F. et al. found that there was no significant correlation between sarcopenia and the incidence of infection and adverse events after posterior lumbar fusion [10]. Furthermore, the study by Brinkmann, E. J. et al. showed that in patients with spinal tumors, there was no significant correlation between sarcopenia and mortality, infection or reoperation after tumor resection [11].

Flexman, A. M. et al. found an unreliable correlation between sarcopenia and adverse health outcomes [2] that may have resulted from differences in the definition of sarcopenia and methods for measuring postoperative outcomes. In addition, the study population and the type of surgery may also be factors that affect the prediction of the incidence of adverse events after spinal surgery [9]. Understanding the importance and relevance of sarcopenia as a risk factor for patients undergoing spinal surgery, as well as better assessing the risk of having a poor prognosis, will help surgeons to optimize treatment. To solve this problem, we conducted this meta-analysis. This meta-analysis systematically reviews previous related studies to evaluate the impact of sarcopenia on the outcome of spinal surgery.

### PICO:

P (Population): Patients ≥18 years of age undergoing spinal surgery
I (Intervention): Patients diagnosed with sarcopenia
C (Comparison): Patients not diagnosed with sarcopenia
O (Outcome): Postoperative outcomes such as adverse events and mortality

## Methods

### Standard protocol approvals, registrations, and patient consent

This study was conducted and reported in accordance with the Preferred Reporting Items for Systematic Review and Meta-Analyses Protocol (PRISMA-P) guidelines [12] and the Assessing the Methodological Quality of Systematic Reviews (AMSTAR) guidelines [13]. The protocol was registered on the website https://www.crd.york.ac.uk/prospero/(Registration number: CRD42023406667).

### Data sources and search strategy

Two independent investigators systematically searched PubMed, Embase and the Cochrane Library from database inception to January 9, 2023, without any language or publication time limitations. We used medical subject headings (MeSH) in our search of PubMed and the Cochrane Library and Embase subject headings (Emtree) in our search of Embase, in addition to free-text words (including closely related words or synonyms) related to spinal surgery, sarcopenia and postoperative complications. The search strategy used the following terms: ("Laminectomy" or "Spin*/surgery" or "Spinal Fusion" or "Spinal Cord Diseases/surgery" or "Laminoplasty" or "Diskectomy" or "Kyphoplasty" or "Cementoplasty" or "Spinal Fractur*" or "Spinal Injur*" or "Spinal Cord Injuries" or "Vertebroplasty" or "Foraminotomy") and ("Sarcopenia" or "Muscular Atrophy" or "Muscle Hypotonia" or "Dystonia" or "Muscle, Skeletal/abnormalities" or "Muscular Disorders, Atrophic" or "Muscle Weakness" or "Muscle Strength" or "Physical Fitness" or "Geriatric Assessment") (eTable 1 in S1 File). Additionally, we manually searched the reference list of previous systematic reviews and meta-analyses for missing papers. When the same cohort was described in multiple articles, only the most recent article or the article that included the largest number of participants was included.

Citations from the initial search were downloaded and merged by using Endnote X9 software, and duplicate records were identified and manually deleted. In accordance with the PICOS guidelines, six investigators independently reviewed the titles and abstracts to identify studies eligible for inclusion. In the event of disagreement, the final decision was made through consultation with two senior reviewers.

### Study selection

Potential studies were considered eligible for inclusion if they met the following preestablished inclusion/exclusion criteria:

1. Participant: patients ≥18 years of age who underwent spinal surgery.
2. Exposure: patients with sarcopenia.
3. Comparator: patients without sarcopenia.
4. Outcome: Adverse events and mortality were the primary outcomes, while other complications (postoperative infection, reoperation, deep venous thrombosis, blood transfusion, readmission) were the secondary outcomes. The effect size was presented as the odds ratio (OR) and 95% confidence interval (CI).
5. Study design: A prospective or retrospective cohort study.

We excluded conference papers, reported individual cases and systematic reviews, or studies that did not provide sufficient data.

### Data extraction

Two researchers used a predesigned spreadsheet to extract data from each included study. Disagreements between reviewers were resolved through discussion or consultation with a third reviewer. The following data were extracted: first author, publication year, types of study, age, average body mass index (BMI), geographic regions, follow-up, sample size, percentage of women, type and site of operation, definitions and measurements of sarcopenia, outcomes, reported OR and adjusted variables.

### Methodological quality assessment

Two authors independently assessed the methodological quality of each qualified study using the Newcastle‐Ottawa Scale (NOS) [14]. The scale covers three domains, including patient representativeness, exposure and outcome determination, and follow-up adequacy. The total score for each study was 9. A score ≥ 8 indicates high quality (low bias risk) [15].

### Statistical analysis

All analyses were conducted using Stata statistical software (version 12.0; Stata, University Station, Texas, USA). Considering the heterogeneity between patient baselines, a fixed-effect model was used [16]. A fully adjusted effect estimate (OR) of the correlation between sarcopenia and the surgical outcomes was used to derive pooled risk estimates described in a forest plot. Heterogeneity between studies was evaluated by the Cochrane Q test and I^2^ statistic. When I^2^ ≥ 50% or P < 0.05, heterogeneity was determined to be statistically significant [16]. To explore the source of heterogeneity in the study, we performed a series of subgroup analyses according to geographical regions (China, USA, and other areas), sample sizes (≤100 individuals, 100–200 individuals or ≥200 individuals), average age (≤65 years, 65–70 years, ≥70 years), average BMI (18.5–24.9 kg/m^2^, 25–29.9 kg/m^2^, ≥30 kg/m^2^), sex (male or female), measurement of sarcopenia (NTPA, SMI or other methods), follow-up period (≤12 months, >12 months), type of surgery (thoracic or lumbar spine) and the quality of the study (low or high). The symmetry of the funnel chart was evaluated visually and combined with Begg’s and Egger’s tests to evaluate publication bias. Begg’s and Egger’s tests and visual examination of funnel plot asymmetry were used to evaluate publication bias. We used Duvall & Tweedie’s trim-and-fill method to further adjust the risk estimate to assess the potential impact of publication bias [17]. Sensitivity analysis was performed by excluding each study to evaluate the stability of the results. For all statistical tests, a P value < 0.05 was considered statistically significant.

## Results

### Literature search

**Fig 1:**
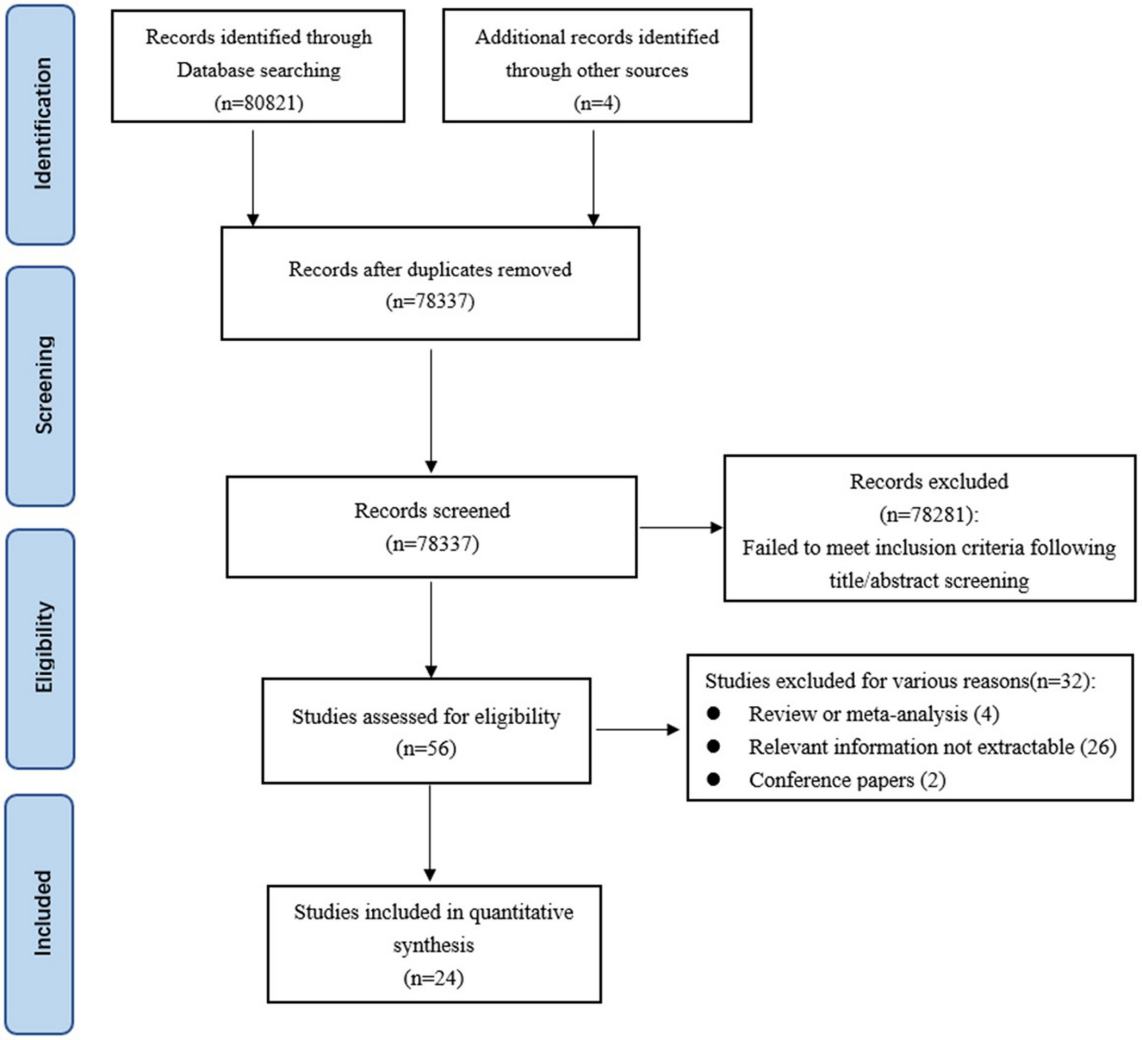
Flowchart of study selection.

A preliminary search yielded 80,821 records and an additional 4 records from other sources. After removing duplicates (n = 2,488), 78,337 titles and abstracts were screened. Of these, 56 articles were selected for full-text review according to the title or abstract. Based on the aforementioned inclusion and exclusion criteria, 32 studies were excluded, leaving 24 studies for inclusion in this meta-analysis (Fig 1).

### Characteristics of studies

Table 1 shows the baseline characteristics of the included studies. The studies included in this review were published between 2015 and 2023, with 70.83% of the studies [3–6, 10, 11, 18–28] published in 2020 or later. Of the 24 studies, 9 studies [3–6, 11, 24, 25, 29, 30] were conducted in the United States, 4 studies [4, 18–20] were conducted in China, and 83.33% (20/24) of the studies included in the analysis were high-quality studies with NOS scores of 8 or higher (**Table 2**). The total sample size of the study was 243,453, the average sample size was 10,144, and the average follow-up time was 23.79 months (range, 3–72 months). Four studies used the skeletal muscle index (SMI) to measure sarcopenia with respect to the measurement standard of muscular dystrophy [18, 20, 26, 31], three studies used the psoas lumbar vertebral index (PLVI) [10, 11, 23], and three studies used the total psoas area/vertebral body area (TPA/VBA) [5, 22, 32]. Our meta-analysis included 2 prospective studies and 22 retrospective studies. The remaining studies measured and assessed sarcopenia using MRI, CT, or working group measurement standards.

**Table 1:** Characteristics of studies included in the meta-analysis.

**First author**	**Year**	**Study Design**	**Region**	**Observation Period**	**Sample size**	**Female%**	**Average age**	**Average BMI kg/m^2^**	**Surgery type**
Akbik, O. S.	2022	Retrospective case series study	USA	2016.1–2021.1	235	65.96	69.60	28.20	Thoracolumbar fusion
Albright, J. A.	2023	Retrospective propensity matched cohort study	USA	2012.1–2019.10	239953	56.53	58.71	NR	Lumbar spine arthrodesis
Barile, F.	2022	Retrospective observational study	Italy	2004–2019	304	50.99	64.00	26.80	Posterior lumbar spinal fusion
Bo, J.	2022	Retrospective study	China	2015.1–2020.1	156	65.38	69.75	26.12	Percutaneous vertebroplasty
Bokshan, S. L.	2016	Retrospective study	USA	2003–2015	46	52.17	72.19	NR	Thoracolumbar spine surgery
Bourassa-Moreau, É	2020	Retrospective cohort study	Canada	2009.1–2016.12	108	47.22	62.50	NR	Emergent surgery for metastatic disease of the spine
Brinkmann, E. J.	2022	Retrospective study	USA	2005–2017	48	47.92	54.00	28.60	Sacral tumor resection
Charest-Morin	2018	Retrospective cohort study	Canada	2009–2013	102	50	73.28	28.18	Elective thoracolumbar surgery
Elie M	2021	Retrospective cohort study	USA	2010–2019	88	26.1	50.93	NR	RCC spinal metastases surgery
Gakhar	2015	Retrospective cohort study	Britain	2009–2013	86	48.9	64.37	NR	Spinal metastasis spinal cord compression
Hirase, T.	2021	Retrospective cohort study	USA	2016.5–2020.2	114	60.53	60.12	30.66	Complex revision surgery for the thoracolumbar spine
Inose, H.	2018	Retrospective anonymized study	Japan	2014.1–2015.12	243	54.32	76.43	NR	Lumbar spinal surgery
Kim, D. U.	2021	Retrospective study	Korea	2019.1–2020.9	85	58.82	74.05	25.12	Thoracic or lumbar spine surgery
Koshimizu	2018	Prospective follow-up study	Japan	2009–2015	171	33.3	71.68	24.14	Cervical Laminoplasty
Li, H.	2021	Retrospective study	China	2016.6–2019.4	69	59.42	60.74	23.28	Lateral lumbar interbody fusion
Li, Z.	2022	Retrospective study	China	2017.8–2020.12	50	56	62.00	26.04	Lumbar decompression surgery
McKenzie, J. C.	2019	Retrospective study	USA	2014.1–2014.12	97	52.58	62.07	31.19	Lumbar fusion
Ohyama, S.	2021	Prospective intervention study	Japan	2016.4–2017.9	60	78.30	77.80	21.90	Balloon kyphoplasty
Ruffilli, A.	2022	Retrospective Study	Italy	2005–2020	308	0.52	63.80	26.50	Posterior Lumbar Fusion
Sakai, Y.	2020	Retrospective case-control study	Japan	2014.4–2017.3	235	42.55	73.20	24.36	Lumbar spinal surgery
Toyoda, H.	2019	Retrospective study	Japan	2015.8–2016.7	130	46.15	76.90	23.35	Minimally invasive lumbar decompression surgery
Wang, H.	2021	Retrospective study	China	2017.2–2018.6	77	53.25	77.38	NR	Percutaneous kyphoplasty
Zakaria, H. M.1	2020	Retrospective case-control study	USA	2002–2012	417	50.00	66.30	NR	Spine surgery
Zakaria, H. M.2	2020	Retrospective cohort study	USA, China	1999–2017	271	42.07	59.72	26.84	Spine surgery for metastatic tumors
**First author**	**Measurements of sarcopenia**		**Sarcopenia definition**			**Outcomes**			**Follow-up period**
Akbik, O. S.	The lowest quartile of PMI values measured at L3 and L4		NR			Discharge disposition,7-day readmission,30-day readmission,90-day readmission, need for revision surgery			72 months
Albright, J. A.	ICD-9 or ICD-10		A gradual, involuntary loss of skeletal muscle mass and strength			Bowel and bladder dysfunction, Dural tear, Incision and drainage, Postoperative infection, Postprocedural fever, Sepsis, Transfusion, Hematoma, Wound dehiscence, AKI, Cardiac arrest, DVT, Pulmonary embolism, Pneumonia, UTI			NR
Barile, F.	PLVI		Syn-drome of progressive and generalized loss of skeletal muscle mass and strength			LOS, Operative time, PLVI, Infection			26.2 months
Bo, J.	SMI		A significant loss of skeletal muscle mass and strength			RBP			12 months
Bokshan, S. L.	The total cross-sectional area of the psoas muscle at the L4 vertebrae		The loss of muscle mass associated with aging and advanced disease			Postoperative complications, Length of hospital stay			62.4 months
Bourassa-Moreau, É	L3-TPA/VBA technique on CT		A progressive loss of skeletal muscle mass, strength, and power			Postoperative occurrence of adverse events			3 months
Brinkmann, E. J.	PLVI		A progressive age‐related loss of skeletal muscle strength and quality			Disease‐free survival, Overall survival, Metastatic disease, Local recurrence, Wound complications, Deep infection, Sacral stress fracture, Ambulatory, Reoperation, Use of pedicled flaps for closure, Positive surgical margins, Mean total operative time, Red blood cells units transfused, Mean MSTS93 score			60 months
Charest-Morin	NTPA		TPA at mid-L3 level adjusted for height			Adverse events, death, discharge home			NR
Elie M	Total muscle mass measurements		NR			Overall mortality			17 months
Gakhar	TPA/VBA		NR			Mortality rates at 1 year			1 year
Hirase, T.	PMI, calculated at the L3 vertebral body measured on preoperative MRI or CT normalized to height^2^ (mm^2^/m^2^).		A generalized decline in skeletal muscle mass and strength			Postoperative anemia requiring transfusion, cardiac complication, sepsis, wound complication, delirium, intra-operative dural tear, AKI, pneumonia, UTI, urinary retention, epidural hematoma, and DVT. Secondary outcome measures were 30-day readmission rates, 30-day reoperation rates, in-hospital mortality rates and postoperative LOS, Discharge disposition			NR
Inose, H.	The ASM index by DXA		Loss of muscle mass, strength, and function related to ageing			JOA score at final follow-up, Recovery rate at final follow-up, VAS score change (Lower back pain), VAS score change (Lower extremity pain), VAS score change (Lower extremity numbness)			13.3 months
Kim, D. U.	Measuring muscle performance or imaging muscle mass.		Loss of muscle mass, strength, and function related to ageing			Postoperative complications, LOS			NR
Koshimizu	SMI		Syndrome characterized by a progressive and all over loss of skeletal muscle mass and force			C2–C7 Lordosis, Angle(Preoperative), C2–C7 Lordosis, Angle(One-year), C2–C7 ROM(Preoperative),C2–C7 ROM(One-year)			1 year
Li, H.	AWGS		Low SMI plus low handgrip strength or low walking speed			ODI, VAS for back pain			16.3 months
Li, Z.	SMI		Age-related decrease in muscle volume and muscle function			Operation time, Intraoperative blood loss, Postoperative drainage volume, Hospitalization stay, VAS score of low back pain, VAS score of sciatica, ODI score			29.7 months
McKenzie, J. C.	Sarcopenia was diagnosed by CT or MRI paraspinal or abdominal muscle CSA.		A progressive loss of skeletal muscle mass and function with resultant disability			ODI, SF-12 Mental, SF-12 Physical, VAS Back Pain			18.3 months
Ohyama, S.	AWGS		An age-related decline in skeletal muscle mass as well as muscle function			Radiological AVF, Reduced ADL, VAS of back pain, PCS of the SF36, MCS of the SF36, Vertebral body wedging angle,°, Vertebral body height, %			6 months
Ruffilli, A.	MRI, PLVI and the Muscle score		Syndrome of progressive and generalized loss of muscle mass and strength			SSI			NR
Sakai, Y.	Both low grip strength and/or low gait speed and low muscle mass		The loss of skeletal muscle mass in the arms and legs			RDQ, EQ5D, SF36 PCS			12 months
Toyoda, H.	The European Working Group on Sarcopenia in Older People definition		Low skeletal muscle index plus low handgrip strength or low walking speed			JOA score, Low back pain, Leg pain, Leg numbness			41 months
Wang, H.	Both grip strength and SMI are below the cut—off values		A progressive and systemic skeletal muscle disease, which mainly refers to the destruction of the shape and function of skeletal muscle			Operation duration, Amount of bleeding, Hospital stay, VAS score one month after operation, ODI score one month after operation, Incidence of refracture within one year			12 months
Zakaria, H. M.	Morpho-metric analysis of the psoas muscle at the L4 vertebral level was performed using previously described methodology.		A decreased reserve to physiologic stressors			Survival			NR
Zakaria, H. M.	Psoas muscle size		Lack of muscle mass			Postoperative new neurological deficit, SSI, SSD, DVT, PE, MI, UTI, PNA, Prolonged intensive care unit stay (>3 d), CVA, Unplanned return to operating room, Unplanned readmission, 30-d mortality, 90-d mortality			3 months

Abbreviations: ADL: Activities of daily living; AKI: acute kidney injury; ASM: Appendicular skeletal muscle mass; AVF: Adjacent vertebral fractures; AWGS: Asia Working Group for Sarcopenia; BMI, body mass index; C2–C7: Second cervical to 7^th^ cervical; CSA: Cross-sectional surface area; CT: Computed tomography; CVA: Cerebrovascular attack; DVT: Deep vein thrombosis; DXA: Dual-energy X-ray absorptiometry; EQ-5D: Euroqol quality of life–5D scale; ICD-10: International Classification of Diseases, 10th Revision; ICD-9: International Classification of Diseases, Ninth Revision; JOA: Japanese Orthopedic Association; L3:The third lumbar; L4: The 4^th^ lumbar; LOS: Length of stay; MCS: Mental component summary; MI: Myocardial infarction; MRI: Magnetic resonance imaging; MSTS93 score: The Musculoskeletal Tumor Society 93 score; NR: None found; NTPA: Normalized total psoas area; ODI: Oswestry Disability Index; PCS: Physical component summary; PE: Pulmonary embolism; PLVI: Psoas lumbar vertebral index; PMI: psoas muscle index; PNA: Pneumonia; RBP: Residual back pain; RCC: Renal cell carcinoma; RDQ: Roland–Morris Disability Questionnaire; ROM: Range of motion; SBRT: stereotactic body radiation therapy; SSI: Surgical site infection; SF-12: Short Form 12 Questionnaire; SF36: Short form 36; SMI: Skeletal muscle index; SSD: Surgical site dehiscence; SVA: Indicates sagittal vertical axis; TPA: Total Psoas Area; USA: United States of America; UTI: Urinary tract infection; VAS: Visual analogue scale; VBA: Vertebral body Area

**Table 2:** Methodological quality score of the included studies based on the Newcastle–Ottawa Scale (NOS) tool.

Author	year	Study Design	Selection				Comparability	Exposure/Outcome			Total Score	Risk of Bias
			Representativeness of cohort *	Selectin of control cohort *	Ascertainment of exposure *	Outcome not present at start *	Comparability of cohorts **	Assessment of outcome *	Length of follow-up *	Adequacy of follow-up *	Total score 9*	
Akbik, O. S.	2022	Retrospective study	*	*	*	*	*	*	*	*	8	Low
Albright, J. A.	2023	Retrospective cohort study	*	*	*	*	**	*		*	8	Low
Barile, F.	2022	Retrospective study	*	*	*	*	**	*	*	*	9	Low
Bo, J.	2022	Retrospective study	*	*	*	*	*	*	*	*	8	Low
Bokshan, S. L.	2016	Retrospective study	*	*	*	*	*	*	*	*	8	Low
Bourassa-Moreau, É	2020	Retrospective cohort study	*	*	*	*	**	*	*	*	9	Low
Brinkmann, E. J.	2022	Retrospective study	*	*	*	*	*	*	*	*	8	Low
Charest-Morin	2018	Retrospective cohort study		*	*	*	*	*		*	6	High
Elie M	2021	Retrospective cohort study	*	*	*	*	**	*	*	*	9	Low
Gakhar	2015	Retrospective cohort study	*	*	*	*	*	*	*	*	8	Low
Hirase, T.	2021	Retrospective cohort study	*	*	*	*	**		*		7	High
Inose, H.	2018	Retrospective study	*	*	*	*	*	*	*	*	8	Low
Kim, D. U.	2021	Retrospective study	*	*	*	*	**	*			7	High
Koshimizu	2018	Prospective follow-up study	*	*	*	*	*	*	*	*	8	Low
Li, H.	2020	Retrospective study	*	*	*	*	**	*	*	*	9	Low
Li, Z.	2022	Retrospective study	*	*	*	*	**	*	*	*	8	Low
McKenzie, J. C.	2019	Retrospective study	*	*	*	*	*	*	*	*	8	Low
Ohyama, S.	2021	Prospective intervention study	*	*	*	*	*	*	*	*	8	Low
Ruffilli, A.	2022	Retrospective Study	*	*	*	*	**	*		*	8	Low
Sakai, Y.	2020	Retrospective study	*	*	*	*	*	*	*	*	8	Low
Toyoda, H.	2019	Retrospective study	*	*	*	*	*	*	*	*	8	Low
Wang, H.	2021	Retrospective study	*	*	*	*	*	*		*	7	High
Zakaria, H. M.1	2020	Retrospective study	*	*	*	*	**	*		*	8	Low
Zakaria, H. M.2	2020	Retrospective study	*	*	*	*	**	*		*	8	Low

### Primary outcome

#### Adverse events

**Fig 2:**
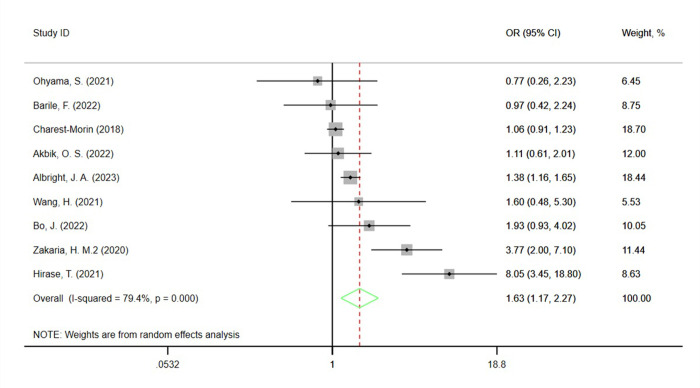
Odds ratio (OR) of the association between sarcopenia and adverse events after spinal surgery.

Nine studies [4–6, 10, 18, 24, 26, 27, 33] reported the association between sarcopenia and adverse events. Overall, in comparison to the nonsarcopenic group, the sarcopenic group had a significantly higher risk of adverse events (OR 1.63, 95% CI 1.17–2.27), with significant heterogeneity observed (I² = 79.4%, P<0.001) (Fig 2).

Subgroup analyses showed that the risk was significantly increased in the subgroups of studies with a female proportion of 50–60%, conducted in China, those focusing on lumbar surgery, average age≤65years and average BMI≥30 kg/m^2^. Additionally, the analysis did not show a significant correlation between sarcopenia and the risk of postoperative complications in subgroups stratified by sample size, sarcopenia measurement methods, average follow-up time, and quality rating. By conducting subgroup analysis stratified by study region, sample size, average age, average BMI, sex, methods for measuring sarcopenia, mean follow-up time, and type of surgery, we found a significant reduction in heterogeneity, indicating that the heterogeneity can be attributed to these variables (Table 3).

**Table 3:** Subgroup analyses of the association between sarcopenia and any adverse events after spinal surgery.

Variables		OR	95% CI	I² (%)	No. studies	P for interaction
Regions						0.004
	USA	2.12	0.89 to 5.06	88.1	3	
	China	2.54	1.51 to 4.29	22.6	3	
	Other	1.05	0.91 to 1.22	0	3	
Sample size						0.004
	≥200	1.55	0.95 to 2.53	71.9	4	
	100–200	2.40	0.77 to 7.45	91.4	3	
	≤100	1.07	0.48 to 2.37	0	2	
Female, %						0.004
	≥60	1.93	0.76 to 4.92	82.3	4	
	50–60	1.36	1.15 to 1.62	0	3	
	≤50	1.92	0.56 to 6.65	93.1	2	
Measurements of sarcopenia						0.004
	NTPA	1.58	0.77 to 3.22	86.3	3	
	SMI	1.83	0.98 to 3.43	0	2	
	Other	1.71	0.74 to 3.96	83.5	4	
Follow-up period						0.097
	>12months	1.06	0.65 to 1.72	0	2	
	≤12months	1.91	0.99 to 3.69	56.1	4	
Surgery type						0.069
	Thoracic or lumbar	2.42	0.65 to 9.01	85.9	3	
	Lumbar	1.73	1.14 to 1.62	0	2	
Average age (years)						0.149
	≤65	2.45	1.07 to 5.58	88.0	4	
	65–70	1.40	0.82 to 2.40	24.2	2	
	≥70	1.06	0.91 to 1.23	0	3	
Average BMI (kg/m^2^)						0.423
	18.5–24.9	0.77	0.26 to 2.26	0	1	
	25–29.9	1.50	0.92 to 2.43	76.1	5	
	≥30	8.05	3.45 to 18.79	0	1	
Quality assessment						0.004
	High quality	0.81	0.50 to 1.30	74.4	6	
	Moderate/low quality	1.18	0.46 to 3.06	74.4	3	

Abbreviations: BMI, body mass index; CI, confidence interval; NTPA, Normalized total psoas area; OR, odd ratio; SMI, Skeletal muscle index; USA, United States of America

**Fig 3:**
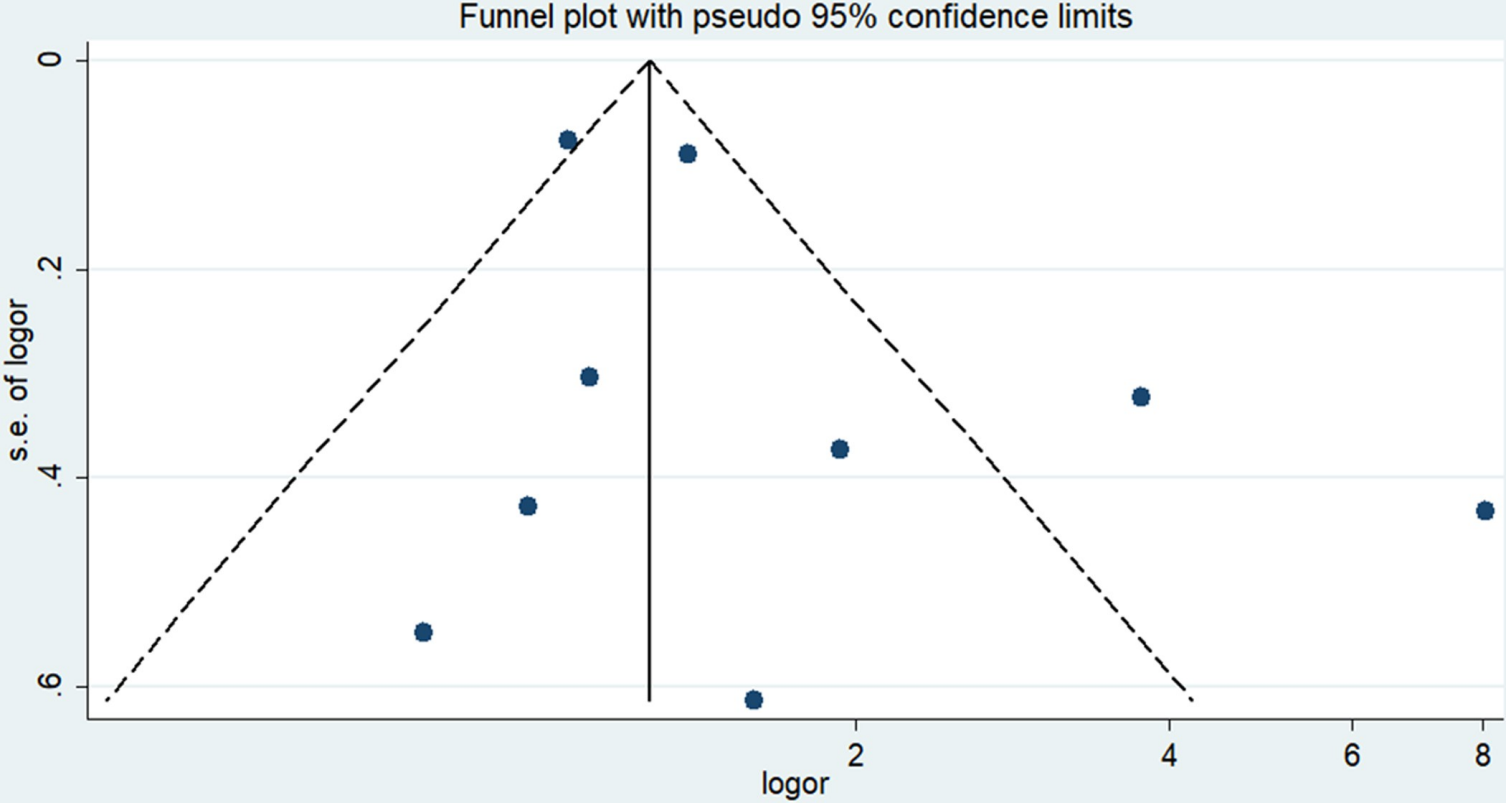
Odds ratio (OR) of the association between sarcopenia and mortality after spinal surgery.

We use sensitivity analysis to analyze the stability of the results. During the analysis, we found that the combined OR did not change significantly due to any individual study (lowest OR = 1.38, 95% CI 1.05–1.80, highest OR = 1.81, 95% CI 1.15–2.85). Visual inspection of the funnel plot showed basic symmetry (Fig 3), which was further validated by Egger’s test (P = 0.199) and Begg’s test (P = 0.917), indicating no publication bias.

#### Mortality

**Fig 4:**
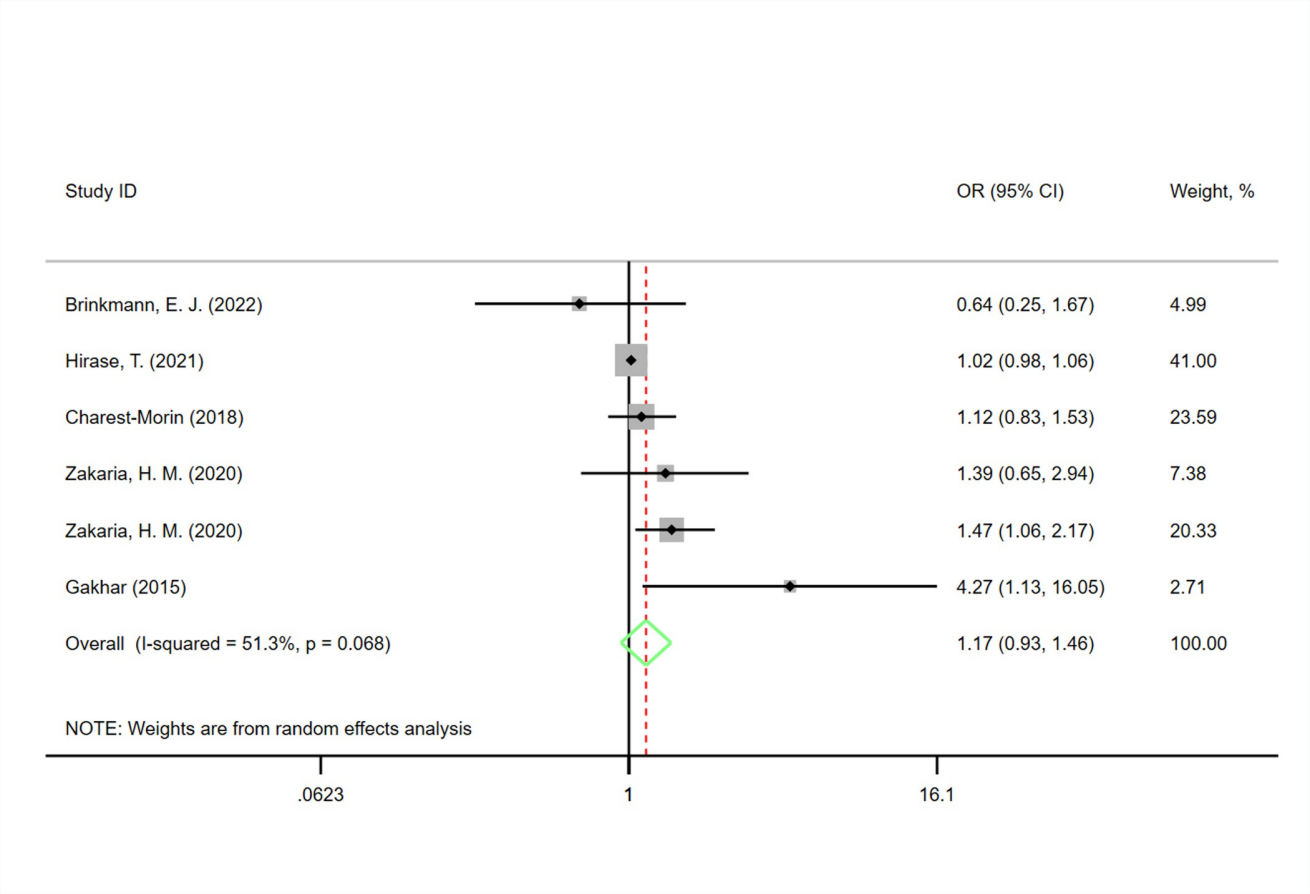
Funnel plots for the meta-analysis of the association between sarcopenia and adverse events after spinal surgery.

Six studies [3–5, 11, 32, 33] reported an association between sarcopenia and postoperative mortality. We did not find significant association between sarcopenia and mortality (OR 1.17, 95% CI 0.93–1.46), and significant heterogeneity was observed (I^2^ = 51.3%, P = 0.068) (Fig 4).

Subgroup analyses showed a significantly increased risk in the subgroups with a sample size ≥200. Furthermore, subgroup analyses of studies conducted in the USA or elsewhere, sex, average age, average BMI, sarcopenia measurement methods, average follow-up time, and quality evaluation did not show a significant association between sarcopenia and postoperative mortality. Heterogeneity was significantly reduced in subgroup analyses of average age, sex and quality evaluation, suggesting that heterogeneity might have originated from these variables (Table 4).

**Table 4:** Subgroup analyses of the association between sarcopenia and mortality after spinal surgery.

Variables		OR	95% CI	I² (%)	No. studies	P for interaction
Region						0.012
	USA	1.33	0.93 to 1.92	69.9	5	
	Other	1.81	0.96 to 3.39	78.7	3	
Sample size						0.012
	≤100	1.56	0.24 to 9.98	80.8	2	
	100–200	1.27	0.94 to 1.72	65.9	4	
	≥200	1.77	1.21 to 2.60	52.3	2	
Female						0.012
	<50	1.94	1.25 to 3.00	43.6	5	
	≥50	1.47	0.92 to 1.36	53.2	3	
Quality assessment						0.012
	High	1.73	1.16 to 2.59	53.1	5	
	Low	1.13	0.88 to 1.45	56.4	3	
Measurements of sarcopenia						0.029
	Psoas muscle	1.62	1.08 to 2.42	55.3	4	
	Thoracolumbar vertebrae	1.12	0.85 to 1.48	58.5	3	
Average BMI (kg/m^2^)						0.001
	25–29.9	1.10	0.84 to 1.44	0	3	
	≥30	1.02	0.98 to 1.06	-	1	
Average age (years)						0.032
	≤60	1.00	0.47 to 2.12	36.3	2	
	60–65	1.78	0.45 to 6.98	77.6	2	
	≥65	1.26	0.97 to 1.64	21.9	2	
Follow-up period						0.040
	<12 months	2.18	1.55 to 3.06	0	2	
	≥12 months	1.56	0.24 to 9.98	80.8	2	

Abbreviations: BMI, body mass index; CI, confidence interval; NTPA: Normalized total psoas area; NR, not reported; OR, odd ratio.

**Fig 5:**
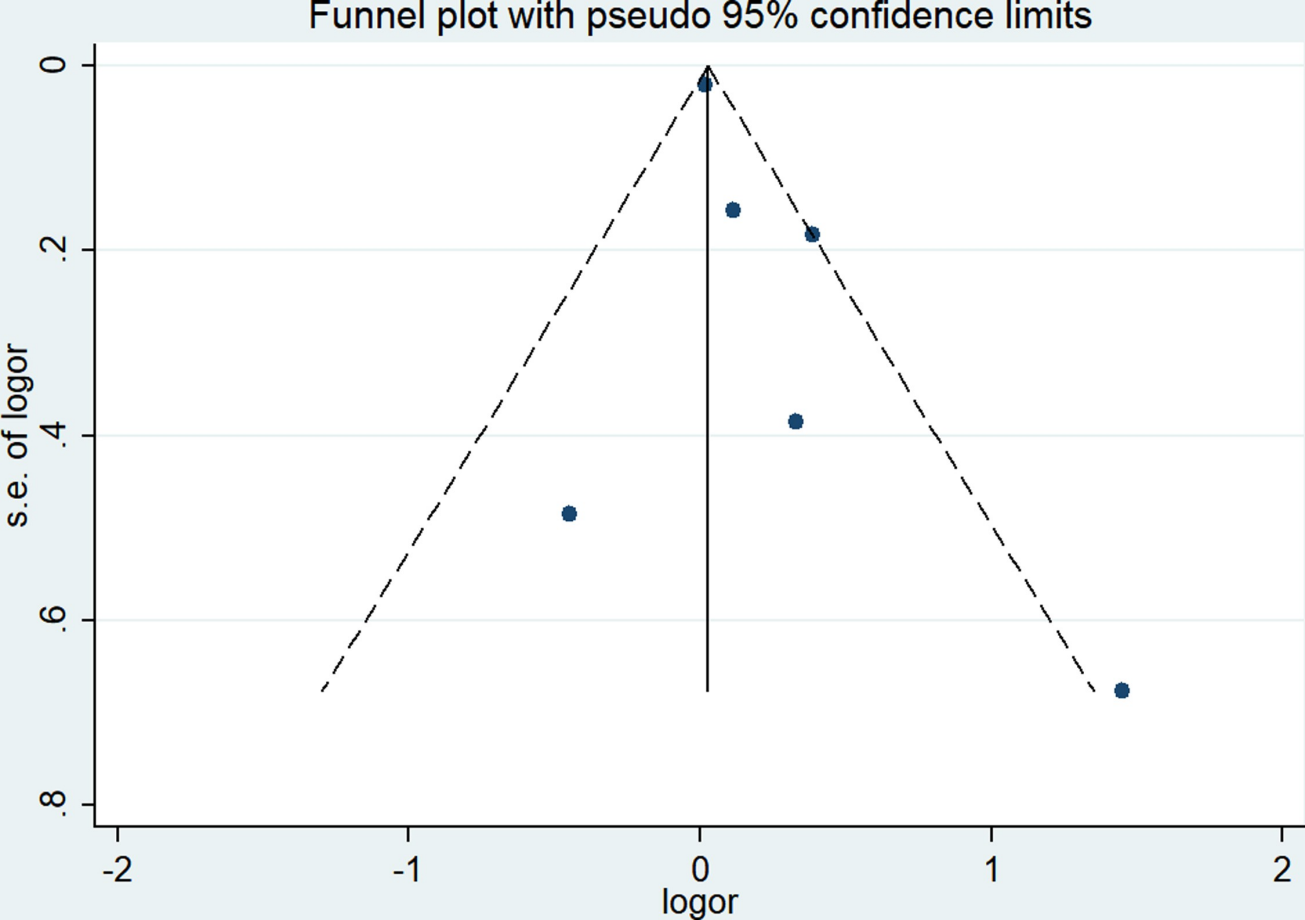
Funnel plots for the meta-analysis of the association between sarcopenia and mortality events after spinal surgery.

Through sensitivity analysis, we found that the combined OR did not change significantly for any individual study (lowest OR = 1.09, 0.87–1.36, highest OR = 1.21, 0.95–1.53). Gross examination of the funnel plot revealed basic symmetry and potential evidence of publication bias (Fig 5), as demonstrated by Begg’s test (P = 0.260) and Egger’s test (P = 0.189). We performed Duvall & Tweedie’s trim and fill method as adjustments to risk estimates to assess the potential effects of publication bias. The results revealed one potentially missing study in the funnel plot region, with an OR value of 1.128 (95% CI 0.877–1.452) after publication bias adjustment, which was similar to the preliminary results.

### Secondary outcomes

We did not find any association between sarcopenia and infection (OR 2.24, 95% CI 0.95–5.26), 30-day reoperation (OR 1.47, 95% CI 0.92–2.36), deep vein thrombosis (OR 1.78, 95% CI 0.69–4.61), postoperative discharge home (OR 0.60, 95% CI 0.26–1.37) or blood transfusion (OR 3.28, 95% CI 0.74–14.64) (S1-S5 Figs in S1 File) (Table 5). There were insufficient studies available to evaluate publication bias for these outcomes (number of studies per outcome < 7); therefore, there was no further evaluation of publication bias for outcomes.

**Table 5:** Odds ratios (ORs) for the association between sarcopenia and secondary outcomes after spinal surgery.

Variables	OR	95% CI	P for association	I², %	P value for heterogeneity	No. of study
Infection	2.24	0.95 to 5.26	0.07	83.40	<0.001	5
30-day reoperation	1.47	0.92 to 2.36	0.11	0	0.41	4
DVT	1.78	0.69 to 4.61	0.23	31.10	0.23	3
Discharge home	0.60	0.26 to 1.37	0.23	84.40	0.02	3
Transfusion	3.28	0.74 to 14.64	0.01	83.20	0.02	2

Abbreviations: CI, confidence interval; DVT, Deep vein thrombosis; OR, odd ratio.

## Discussion

### Principal findings

This meta-analysis systematically reviewed data from 24 cohort studies and analyzed the relationship between sarcopenia and other postoperative outcomes of spinal surgery. Our meta-analysis found a significant correlation between sarcopenia and the risks of postoperative adverse events and mortality. However, we did not find a significant correlation between sarcopenia and the risks of infection, 30-day reoperation, deep vein thrombosis, return home after surgery, or blood transfusion.

### Potential mechanisms

The increase in the incidence of adverse events after spinal surgery in patients with sarcopenia is attributable to a decrease in protein reserves, disruption of muscle protein homeostasis leading to a decrease in muscle mass, and an increase in systemic inflammation [34, 35]. Li Ziquan et al. found that the mechanism of poor prognosis in patients with sarcopenia was multifactorial, including muscle protein homeostasis imbalance, reduced reserves to respond to stress stressful events, and an enhanced systemic inflammatory response [20, 36, 37]. The investigation found that there are many muscle tissue factors that regulate bone tissue activity, and sarcopenia can affect various regulatory factors related to bone remodeling, such as insulin-like growth factor (IGF-1), fibroblast growth factor (FGF-2), interleukins (IL-6, IL-15), myostatin, bone glycine, and irisin. Therefore, sarcopenia is related to a decrease in bone density [38–40]. A reduced bone mineral density is an important factor influencing the occurrences of falls and fractures and may also be a factor that contributes to an increase in the incidence of postoperative adverse events. In addition, sarcopenia is associated with hormonal imbalances and increased cytokine activity, which can lead to physical decline [38–41], and it may be related to an increase in the incidence of adverse events and mortality in patients after surgery. Zakaria, H.M. et al. found that sarcopenia may reflect the "cachexia" phenotype, which is a result of metabolic abnormalities in patients with advanced cancer and a potential cause of increased mortality after spinal surgery in sarcopenia patients [4, 42, 43].

A study found that the level of Runx2, a transcription factor essential to osteocyte maturation in patients with sarcopenia, was decreased, which was associated with a decrease in bone mineral density [44]. According to Mechanostat theory, the direct mechanical stimulation of bone caused by muscle contraction can promote osteogenesis, but this part of stimulation is lacking in sarcopenia patients [45]. In addition, sarcopenia and osteoporosis share many common pathways, such as sensitivity to decreased secretion of anabolic hormones, increased activity of inflammatory cytokines, and release of anabolic or catabolic molecules (muscle factors and bone factors) from skeletal muscle or osteocytes, resulting in reduced physical activity [46–48]. In the Ma et al study, it is found that muscle can affect bone mineral density through some physical mechanisms or the release of biological factors, and lean body weight may be a factor affecting bone mineral density [45]. To sum up, sarcopenia is likely to be a factor leading to the decrease of bone mineral density. In addition, there is growing evidence that bones and muscles can secrete various cytokines that regulate each other, including myostatin, irisin, interleukin 6, osteocalcin, RANKL, and osteoprotegerin [49]. In patients with sarcopenia, the reduced muscle activity may lead to relatively inadequate secretion of certain cytokines or hormones vital for metabolism and muscle function. These hormones play an important role in the human body, and the relative deficiency of these important hormones may be an important cause of adverse events after spinal surgery in patients with sarcopenia.

### Comparison with other studies

When investigating the relationship between sarcopenia and mortality after spinal surgery, we found three similar systematic reviews [9, 50, 51], all of which indicated an increased risks of sarcopenia and mortality after spinal surgery. However, two of them did not find a significant correlation between sarcopenia and the incidence of adverse events after spinal surgery [9, 51]. In addition, the above studies have certain limitations due to reporting bias, insufficient subgroup analysis, inconsistent measurement standards for sarcopenia, etc. We used Duvall & Tweedie’s pruning and filling methods to adjust the risk estimates to evaluate the potential impact of publication bias and conducted a series of subgroup analyses to explore the heterogeneity of the study. This study is currently the largest and most comprehensive study investigating the relationship between sarcopenia and different outcomes after spinal surgery for nearly 240,000 participants.

### Implications

In this study, the approximate risk of various complications after spinal surgery are estimated in patients with sarcopenia to provide guide future clinical practice. Preoperative measurement and effective management of skeletal muscle mass in patients have important clinical significance in preventing postoperative death and adverse events. At present, clinical doctors do not realize the importance of managing sarcopenia before spinal surgery and thus have not established standardized treatment for sarcopenia patients before surgery. This study provides new evidence-based medical evidence for clinical doctors to treat such patients. For patients with sarcopenia, providing appropriate and effective treatment before surgery will help to reduce the occurrence of postoperative complications.

### Strengths

The current meta-analysis has the following strengths. First, we comprehensively searched the literature in three major databases, PubMed, Cochrane Library, and Embase, using MeSH/Emtree and free text terms and developed a comprehensive database search strategy that was not limited by date or language. As a result, we found original articles that met the inclusion criteria to avoid publication bias and improve the reproducibility of the results. Second, we adhered to the PRISMA guidelines and used the NOS scale to provide complete, informative, and transparent scoring criteria for the included studies. Third, we used several methods to fully test the stability of the results, including sensitivity analysis and subgroup analysis. Finally, by using the trim-and-fill method to adjust the summary estimation based on publication bias, we found that the results were consistent with the initial results.

### Limitations

Our research still presents some potential limitations. First, we found that there was some interstudy heterogeneity in the occurrences of postoperative mortality and adverse events, possibly due to a lack of standardization in the measurement of sarcopenia and differences in the baseline characteristics of the study cohort. However, we conducted further analysis to evaluate the source of this heterogeneity, and the adjusted results were consistent with the initial results. The subgroup analysis found that interstudy heterogeneity had little impact on the final results. Second, our conclusions were based on the data of a retrospective cohort study, so we cannot infer the causal relationship between sarcopenia and the incidences of adverse events and mortality after spinal surgery, which is also an inherent limitation of meta-analyses. Third, we also found that the impact of some risk factors was estimated near the border, with a confidence interval between 0.95 and 5.26 (such as infection). These findings need to be verified in a large prospective cohort study. Finally, at present, the diagnosis of sarcopenia is not unified, and there are no restrictions on the diagnosis of sarcopenia in our study. There are some differences between the different diagnostic methods of sarcopenia among the included studies, and these differences will affect our correct evaluation of the impact of sarcopenia on spinal surgery.

## Conclusions

The current meta-analysis showed that patients with sarcopenia have increased risks of adverse events and mortality after spinal surgery. However, these results must be carefully interpreted because the number of studies included is small and the studies are significantly different. These findings may help to increase the clinicians’ awareness of the risks that are concerns patients with sarcopenia to improve their prognosis.

## Supporting information

S1 Checklist PRISMA 2020 checklist. (DOCX)

S1 File (DOCX)
