# Supplementary material for: The impact of sarcopenia on the incidence of postoperative outcomes following spine surgery: Systematic review and meta-analysis
Source: PLoS One. 2024 Aug 26;19(8):e0302291. doi: 10.1371/journal.pone.0302291 (PMC11346935; doi:10.1371/journal.pone.0302291)

**Supplemental data**

**eTable1:** General Search Strategies for PubMed, Embase and Cochrane Library.

**Text S1 Search strategy**

**Database: Pubmed from inception to Present> (Search date:** **January 9，2023)**

**Search Strategy:**

--------------------------------------------------------------------------------

***Spine surgery terms（P）:***

#1 "Laminectomy"[Mesh]

#2 "Spin*/surgery"[Mesh]

#3 "Spinal Fusion"[Mesh]

#4 "Spinal Cord Diseases/surgery"[Mesh]

#5 "Laminoplasty"[Mesh]

#6 "Diskectomy"[Mesh]

#7 "Kyphoplasty"[Mesh]

#8 "Cementoplasty"[Mesh]

#9 "Spinal Fractur*"[Mesh]

#10 "Spinal Injur*"[Mesh]

#11 "Spinal Cord Injuries"[Mesh]

#12 "Vertebroplasty"[Mesh]

#13 "Foraminotomy"[Mesh]

#14 (interbody fusion or lumbar fusion or spinal fusion or spinal instrument or spin* surgery or lumbar degenerative disease or spondylo* or lumbar vertebr* or cervical fusion or cervical fixation or spinal stenosis or laminoto* or PVP or spinal fractur* or transverse process fractur* or spinous process fractur* or burst fractur* or vertebral fractur* or cervical fractur* or thoracic fractur* or lumbar fractur* or facet fractur* or endplate fractur* or spinal injur* or TLIF or PLIF or ALIF or LLIF or XLIF or OLIF or pedicle subtraction osteotomy or vertebral column resection or anterior column realignment or thoracolumbar or sacra or Smith-Petersen osteotomy) [Title/Abstract]

#15 #1-14/or

***Sarcopenia terms(I):***

#16 "Sarcopenia"[Mesh]

#17 "Muscular Atrophy"[Mesh]

#18 "Muscle Hypotonia"[Mesh]

#19 "Dystonia"[Mesh]

#20 "Muscle, Skeletal/abnormalities"[Mesh]

#21 "Muscular Disorders, Atrophic"[Mesh]

#22 "Muscle Weakness"[Mesh]

#23 "Muscle Strength"[Mesh]

#24 "Physical Fitness"[Mesh]

#25"Geriatric Assessment"[Mesh]

#26 (sarcopeni* or muscle mass or muscle function difici* or muscle wasting or muscle atrophy or myopenia or muscle depletion or presarcopenia or low muscle count or limb dystonia or muscle senescence or skeletal muscle or EWGSOP) [Title/Abstract]

#27 #16-26/or

***Outcome terms（O）:***

#28 "Postoperative Complications"[Mesh]

#29 "Mortality"[Mesh]

#30 "Surgical Wound Infection"[Mesh]

#31 "Reoperation"[Mesh]

#32 "Infections"[Mesh]

#33 "Length of Stay"[Mesh]

#34 "Body Mass Index"[Mesh]

#35 "Walking Speed"[Mesh]

#36 "Venous Thrombosis"[Mesh]

#37 "Emergence Delirium"[Mesh]

#38 "Sexual Dysfunction, Physiological"[Mesh]

#39 "Urinary Tract Infections"[Mesh]

#40 "Trauma, Nervous System"[Mesh]

#41 "Urinary Retention"[Mesh]

#42"Frailty"[Mesh]

#43 "Asthenia"[Mesh]

#44 "Morbidity"[Mesh]

#45 "Cardiovascular Diseases"[Mesh]

#46 (severe complications or morbidity or death or operative time or complicatio*, postoperative or patient reported outcomes,mv or revision surger* or length of stay or LOS or 90-day readmission or skeletal muscle mass or SMI or BMI or gait speed or coronary adj2 disease or angina or postoperative delirium or delirium or confusion or transient mental disorder or dementia or cognitive disorders or VTE or CVT or CVST or BMD)[Title/Abstract]

#47 #28-46/or

***Final search results: Combining Spine surgery and Sarcopenia and Outcome:***

#48 #15 and #27 and #47 (3759)

**Text S2 Search strategy**

**Database: EMBASE from inception to Present> (Search date: January 9，2023)**

**Search Strategy:**

--------------------------------------------------------------------------------

***Spine surgery terms（P）:***

#1 ‘laminectomy’/exp

#2 ‘spine surgery’/exp

#3 ‘spine fusion’/exp

#4 ‘spinal cord diseases’/exp

#5 ‘laminoplasty’/exp

#6 ‘discectomy’/exp

#7 ‘kyphoplasty’/exp

#8 ‘cementoplasty’/exp

#9 ‘spine fracture’/exp

#10 ‘spine injury’/exp

#11 ‘spinal cord injury’/exp

#12 ‘percutaneous vertebroplasty’/exp

#13 ‘foraminotomy’/exp

#14 (‘interbody fusion’ or ‘lumbar fusion’ or ‘spinal fusion’ or ‘spinal instrument’ or ‘spin* surgery’ or ‘lumbar degenerative disease’ or spondylo* or ‘lumbar vertebr*’ or ‘cervical fusion’ or ‘cervical fixation’ or ‘spinal stenosis’ or laminoto* or PVP or ‘spinal fractur*’ or ‘transverse process fractur*’ or ‘spinous process fractur*’ or ‘burst fractur*’ or ‘vertebral fractur*’ or ‘cervical fractur*’ or ‘thoracic fractur*’ or ‘lumbar fractur*’ or ‘facet fractur*’ or ‘endplate fractur*’ or ‘spinal injur*’ or TLIF or PLIF or ALIF or LLIF or XLIF or OLIF or ‘pedicle subtraction osteotomy’ or ‘vertebral column resection’ or ‘anterior column realignment’ or thoracolumbar or sacra or ‘Smith-Petersen osteotomy’): ab,ti

#15 #1-14/or

***Sarcopenia terms(I):***

#16 ‘sarcopenia’/exp

#17 ‘muscle atrophy’/exp

#18 ‘muscle hypotonia’/exp

#19 ‘dystonia’/exp

#20 ‘skeletal muscle’/exp

#21 ‘muscle weakness’/exp

#22 ‘muscle strength’/exp

#23 ‘fitness’/exp

#24 ‘geriatric assessment’/exp

#25(sarcopeni* or ‘muscle mass’ or ‘muscle function difici*’ or ‘muscle wasting’ or ‘muscle atrophy’ or myopenia or ‘muscle depletion’ or presarcopenia or ‘low muscle count’ or ‘limb dystonia’ or ‘muscle senescence’ or ‘skeletal muscle’ or EWGSOP): ab,ti

#26 #16-25/or

***Outcome terms（O）:***

#27 ‘postoperative complication’/exp

#28 ‘mortality’/exp

#29 ‘surgical infection’/exp

#30 ‘reoperation’/exp

#31 ‘infection’/exp

#32 ‘length of stay’/exp

#33 ‘body mass’/exp

#34 ‘walking speed’/exp

#35 ‘vein thrombosis’/exp

#36 ‘emergence agitation’/exp

#37 ‘sexual dysfunction’/exp

#38 ‘urinary tract infections’/exp

#39 ‘nervous system injury’/exp

#40 ‘urinary retention’/exp

#41 ‘frailty’/exp

#42 ‘asthenia’/exp

#43 ‘morbidity’/exp

#44 ‘cardiovascular disease’/exp

#45 (‘severe complications’ or morbidity or death or ‘operative time’ or ‘complicatio*, postoperative’ or ‘patient reported outcomes,mv’ or ‘revision surger*’ or ‘length of stay’ or LOS or ‘90-day readmission’ or ‘skeletal muscle mass’ or SMI or BMI or ‘gait speed’ or ‘coronary adj2 disease’ or angina or ‘postoperative delirium’ or delirium or confusion or ‘transient mental disorder’ or dementia or ‘cognitive disorders’ or VTE or CVT or CVST or BMD): ab,ti

#46 #27-45/or

***Final search results: Combining Spine surgery and Sarcopenia and Outcome:***

#47 #15 and #26 and #46 (76278)

**Text S3 Search strategy**

**Database: Cochrane Library from inception to Present> (Search date: January 9，2023)**

**Search Strategy:**

--------------------------------------------------------------------------------

***Spine surgery terms（P）:***

#1 MeSH descriptor: [Laminectomy] explode all trees

#2 MeSH descriptor: [Spinal Fusion] explode all trees

#3 MeSH descriptor: [Spinal Cord Diseases] explode all trees

#4 MeSH descriptor: [Laminoplasty] explode all trees

#5 MeSH descriptor: [Diskectomy] explode all trees

#6 MeSH descriptor: [Kyphoplasty] explode all trees

#7 MeSH descriptor: [Cementoplasty] explode all trees

#8 MeSH descriptor: [Spinal Fractures] explode all trees

#9 MeSH descriptor: [Spinal Injuries] explode all trees

#10 MeSH descriptor: [Spinal Cord Injuries] explode all trees

#11 MeSH descriptor: [Vertebroplasty] explode all trees

#12 MeSH descriptor: [Foraminotomy] explode all trees

#13 ((interbody fusion or lumbar fusion or spinal fusion or spinal instrument or spin* surgery or lumbar degeneratve disease or spondylo* or lumbar vertebr* or cervical fusion or cervical fixation or spinal stenosis or laminoto* or PVP or spinal fractur* or transverse process fractur* or spinous process fractur* or burst fractur* or vertebral fractur* or cervical fractur* or thoracic fractur* or lumbar fractur* or facet fractur* or endplate fractur* or spinal injur* or TLIF or PLIF or ALIF or LLIF or XLIF or OLIF or pedicle subtraction osteotomy or vertebral column resection or anterior column realignment or thoracolumbar or sacra or Smith-Petersen osteotomy)): ti,ab,kw (Word variations have been searched)

#14 #1 or #2 or #3 or #4 or #5 or #6 or #7 or #8 or #9 or #10 or #11 or #12 or #13

***Sarcopenia terms(I):***

#15 MeSH descriptor: [Sarcopenia] explode all trees

#16 MeSH descriptor: [Muscular Atrophy] explode all trees

#17 MeSH descriptor: [Muscle Hypotonia] explode all trees

#18 MeSH descriptor: [Dystonia] explode all trees

#19 MeSH descriptor: [Muscle, Skeletal] explode all trees

#20 MeSH descriptor: [Muscular Disorders, Atrophic] explode all trees

#21 MeSH descriptor: [Muscle Weakness] explode all trees

#22 MeSH descriptor: [Muscle Strength] explode all trees

#23 MeSH descriptor: [Physical Fitness] explode all trees

#24 MeSH descriptor: [Geriatric Assessment] explode all trees

#25 ((sarcopeni* or muscle mass or muscle function difici* or muscle wasting or muscle atrophy or myopenia or muscle depletion or presarcopenia or low muscle count or limb dystonia or muscle senescence or skeletal muscle or EWGSOP)): ti,ab,kw (Word variations have been searched)

#26 #15 or #16 or #17 or #18 or #19 or #20 or #21 or #22 or #23 or #24 or #25

***Outcome terms（O）:***

#27 MeSH descriptor: [Postoperative Complications] explode all trees

#28 MeSH descriptor: [Mortality] explode all trees

#29 MeSH descriptor: [Surgical Wound Infection] explode all trees

#30 MeSH descriptor: [Reoperation] explode all trees

#31 MeSH descriptor: [Infections] explode all trees

#32 MeSH descriptor: [Length of Stay] explode all trees

#33 MeSH descriptor: [Body Mass Index] explode all trees

#34 MeSH descriptor: [Walking Speed] explode all trees

#35 MeSH descriptor: [Venous Thrombosis] explode all trees

#36 MeSH descriptor: [Emergence Delirium] explode all trees

#37 MeSH descriptor: [Sexual Dysfunction, Physiological] explode all trees

#38 MeSH descriptor: [Urinary Tract Infections] explode all trees

#39 MeSH descriptor: [Trauma, Nervous System] explode all trees

#40 MeSH descriptor: [Urinary Retention] explode all trees

#41 MeSH descriptor: [Frailty] explode all trees

#42 MeSH descriptor: [Asthenia] explode all trees

#43 MeSH descriptor: [Morbidity] explode all trees

#44 MeSH descriptor: [Cardiovascular Diseases] explode all trees

#45 ((severe complications or morbidity or death or operative time or complicatio*, postoperative or patient reported outcomes,mv or revision surger* or length of stay or LOS or readmission or skeletal muscle mass or SMI or BMI or gait speed or coronary adj2 disease or angina or postoperative delirium or delirium or confusion or transient mental disorder or dementia or cognitive disorders or VTE or CVT or CVST or BMD)): ti,ab,kw (Word variations have been searched)

#46 #27 or #28 or #29 or #30 or #31 or #32 or #33 or #34 or #35 or #36 or #37 or #38 or #39 or #40 or #41 or #42 or #43 or #44 or #45

***Final search results: Combining Spine surgery and Sarcopenia and Outcome:***

#47 #14 and #26 and #46 (857)

**Fig 1.** Odds ratio (OR) for association between sarcopenia and infection after spinal surgery.


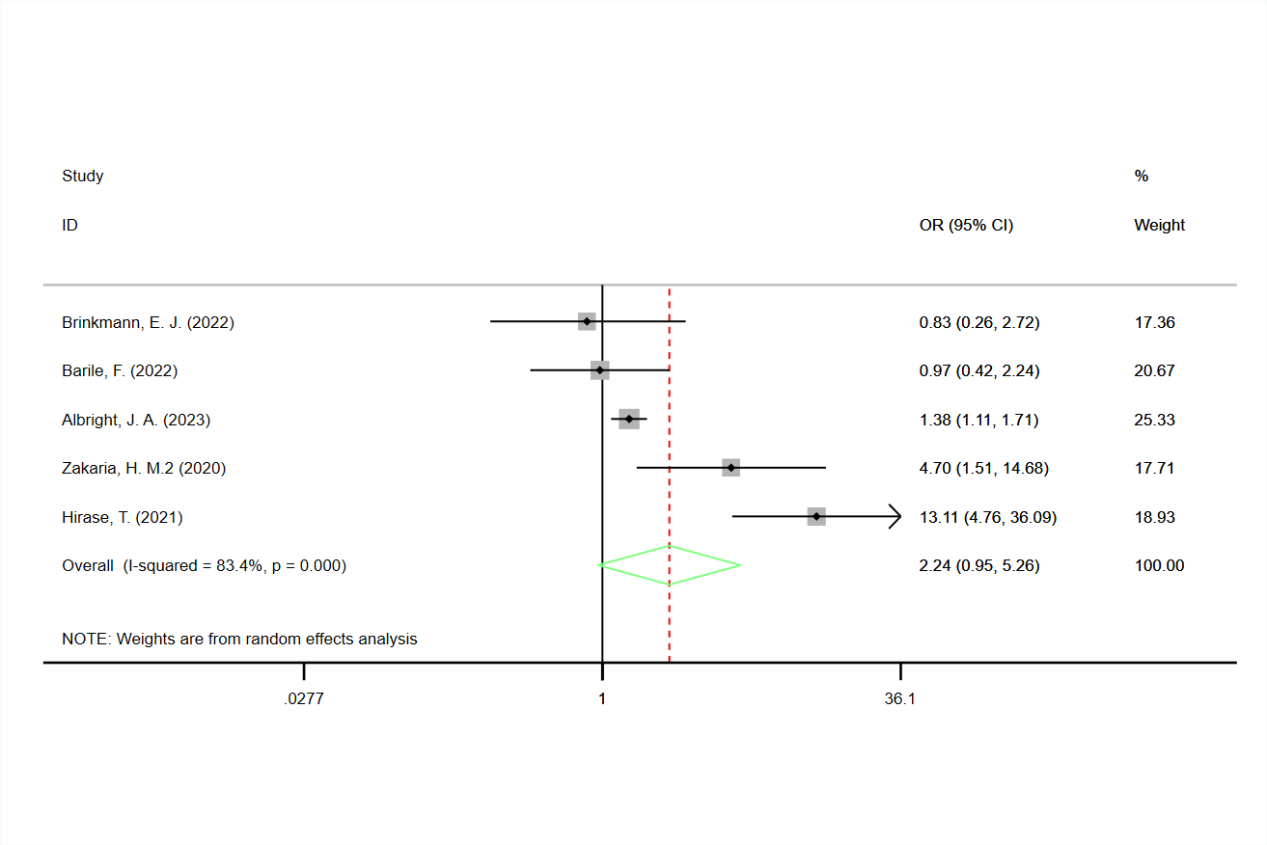


**Fig 2.** Odds ratio (OR) for association between sarcopenia and 30-day reoperation after spinal surgery.


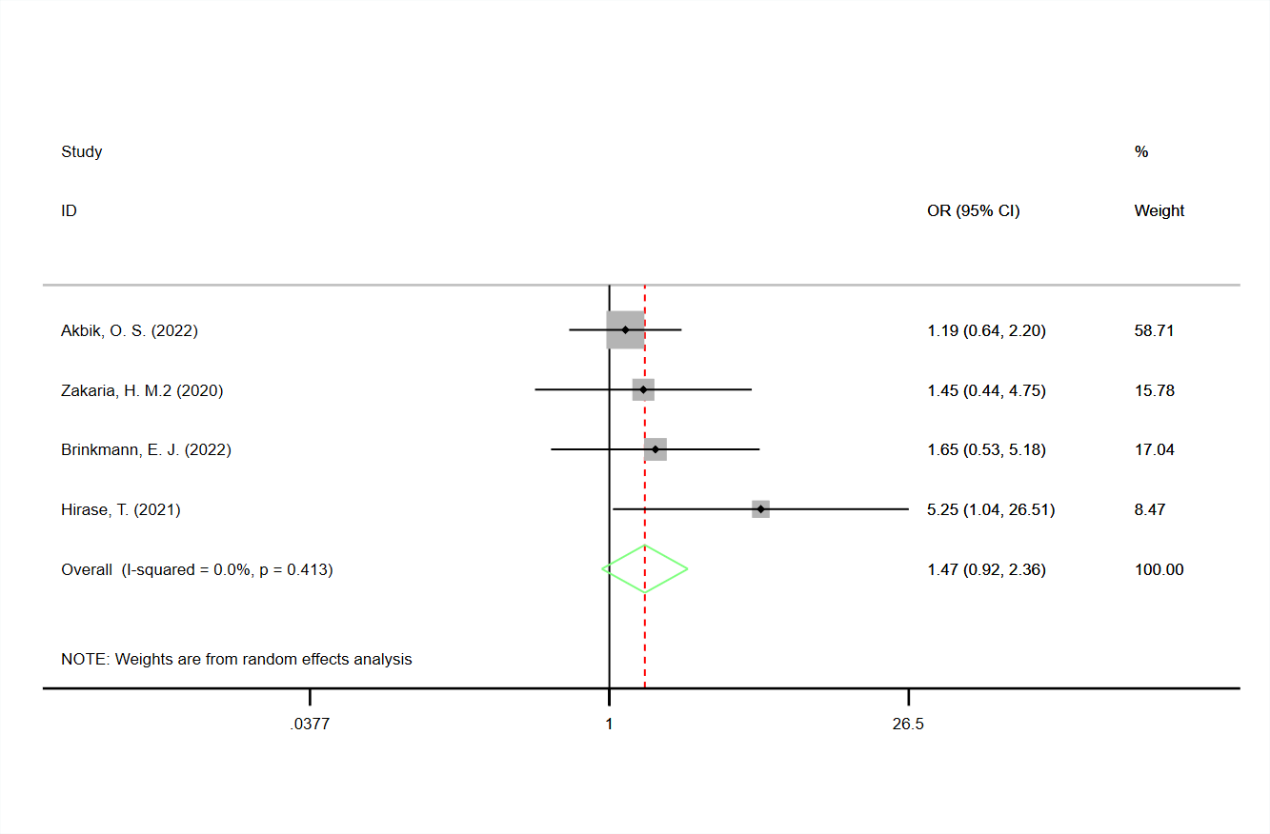


**Fig 3.** Odds ratio (OR) for association between sarcopenia and deep vein thrombosis after spinal surgery.


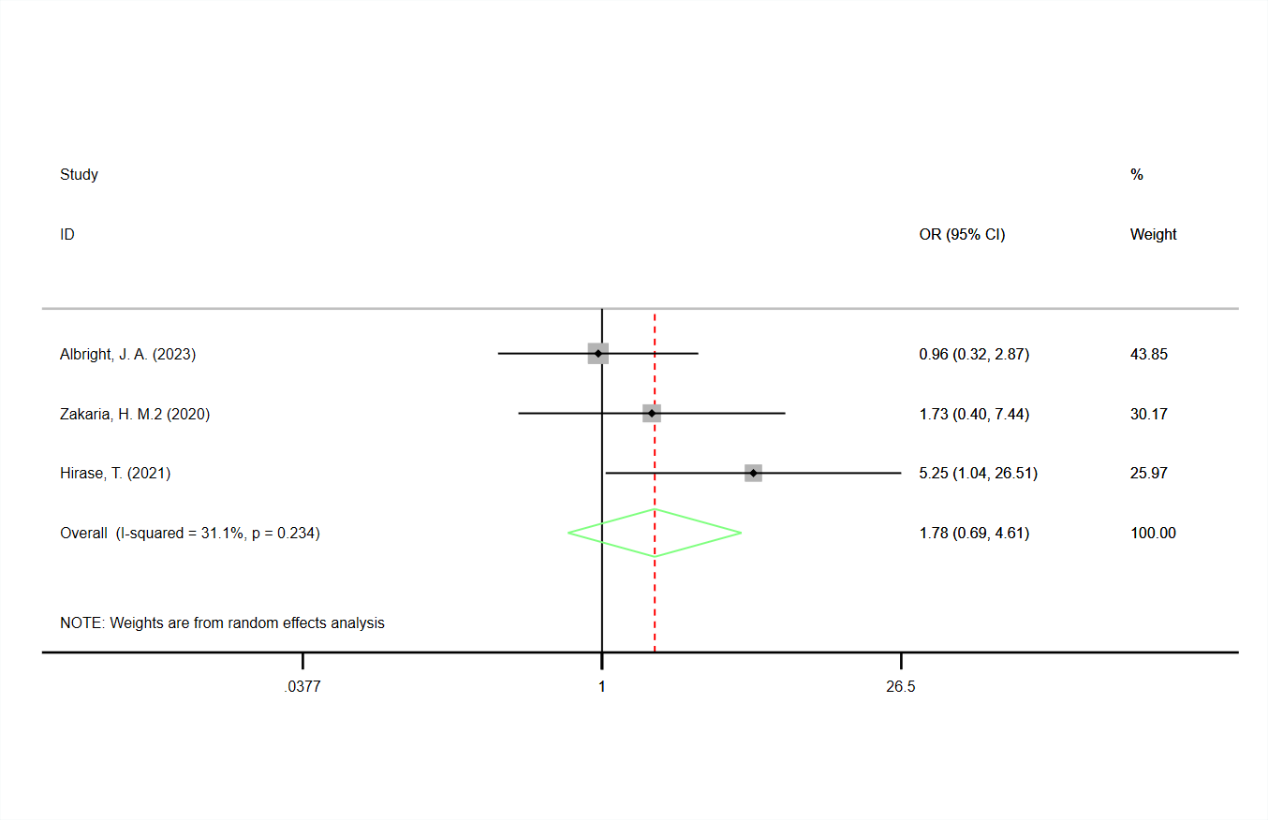


**Fig 4.** Odds ratio (OR) for association between sarcopenia and postoperative discharge home after spinal surgery.


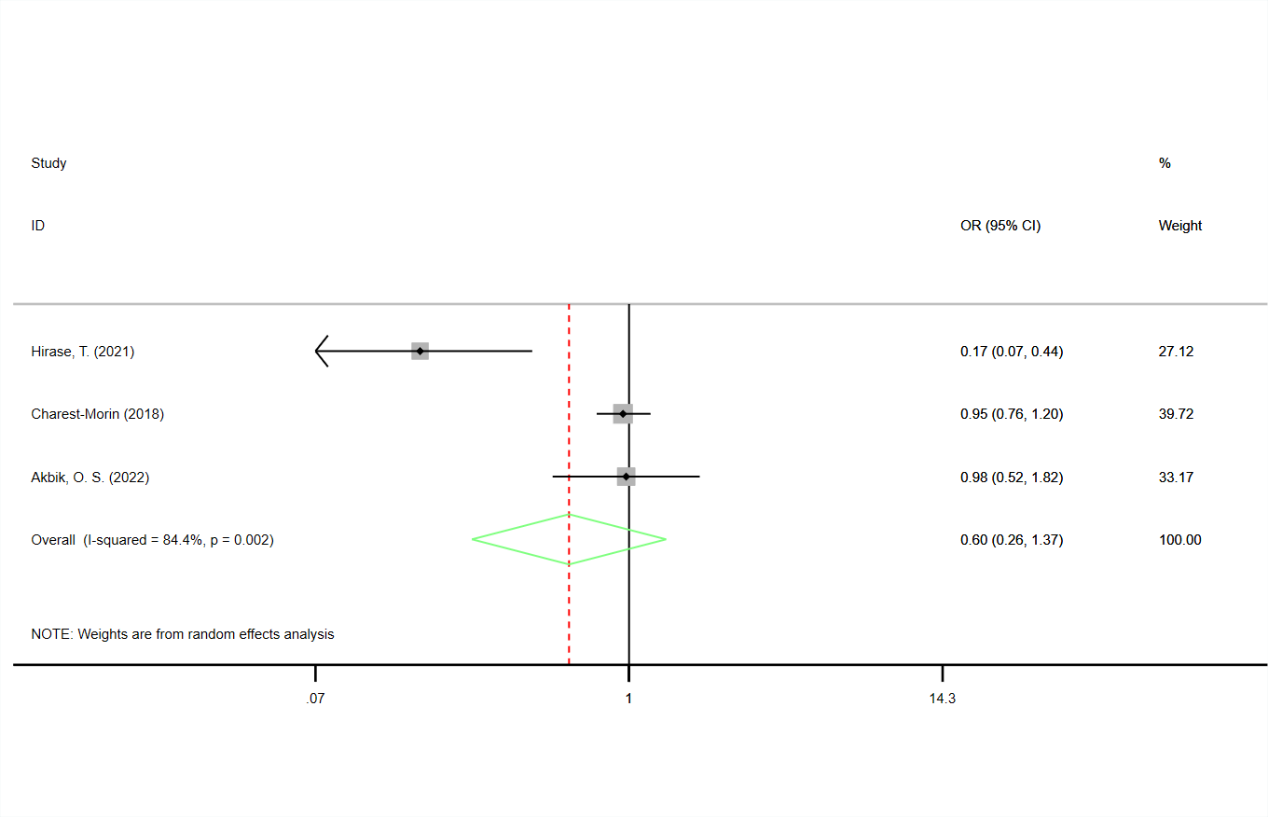


**Fig 5.** Odds ratio (OR) for association between sarcopenia and blood transfusion after spinal surgery.


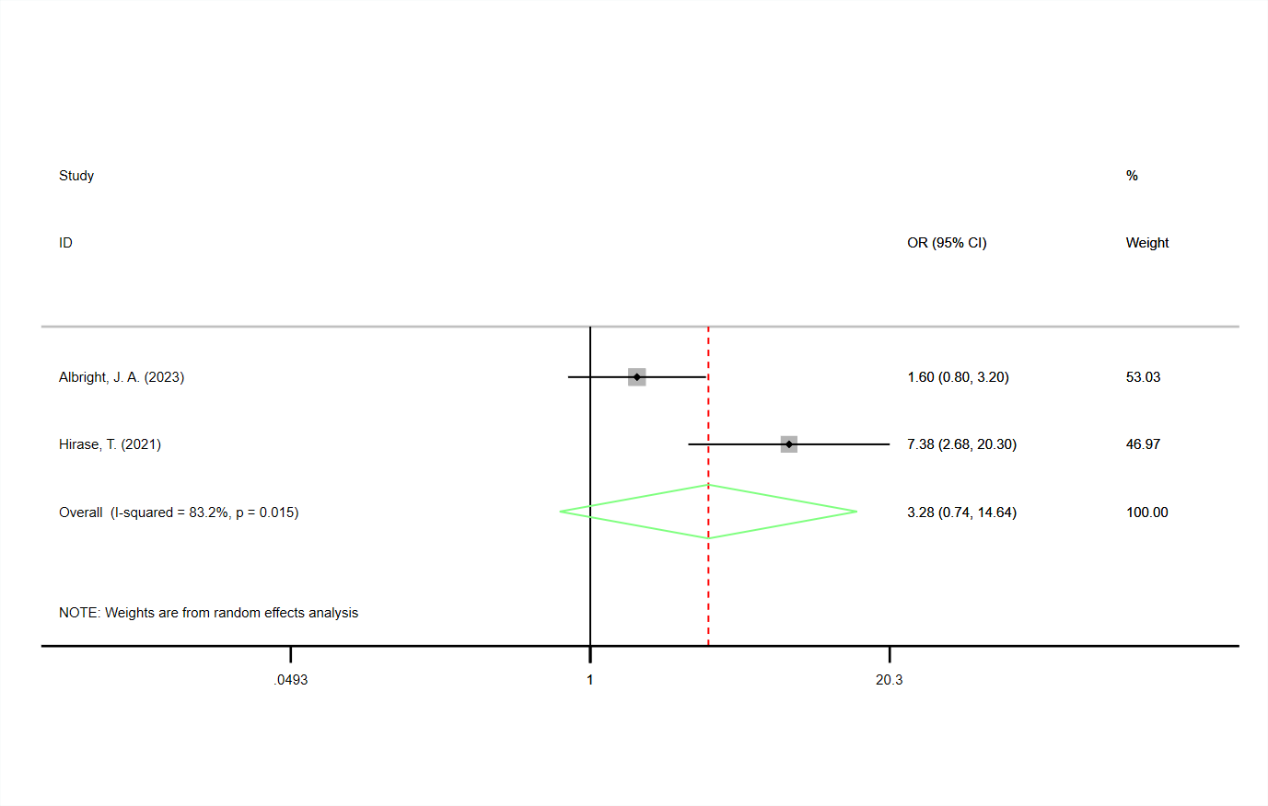

Supplement: S1 File — (DOCX) [file pone.0302291.s002.docx]
